# IL-23/IL-17 axis levels in gingival crevicular fluid of subjects with periodontal disease: a systematic review

**DOI:** 10.1186/s12903-024-04077-0

**Published:** 2024-03-02

**Authors:** Mario Alberto Alarcón-Sánchez, Celia Guerrero-Velázquez, Julieta Sarai Becerra-Ruiz, Ruth Rodríguez-Montaño, Anna Avetisyan, Artak Heboyan

**Affiliations:** 1 Biomedical Science, Faculty of Chemical-Biological Sciences, Autonomous University of Guerrero, Chilpancingo de los Bravo, Guerrero 39090, Mexico; 2 Research Institute of Dentistry, Department of Integrated Dentistry Clinics, University of Guadalajara (CUCS-UdeG), 950 Sierra Mojada, Guadalajara 44340, Jalisco Mexico; 3 Institute of Research of Bioscience, University Center of Los Altos, University of Guadalajara, Tepatitlán de Morelos 47600, Jalisco, Mexico; 4 Department of Health and Illness as an Individual and Collective Process, University Center of Tlajomulco, University of Guadalajara (CUTLAJO-UdeG), Tlajomulco, Santa Fé Highway Km 3.5 No. 595, Lomas de Tejeda, Tlajomulco de Zuñiga 45641, Jalisco Mexico; 5 Department of Therapeutic Stomatology, Faculty of Stomatology, Yerevan State Medical University after Mkhitar Heratsi, Str. Koryun 2, Yerevan, 0025 Armenia; 6 Department of Research Analytics, Saveetha Dental College and Hospitals, Saveetha Institute of Medical and Technical Sciences, Saveetha University, Chennai, 600 077 India; 7 Department of Prosthodontics, Faculty of Stomatology, Yerevan State Medical University after Mkhitar Heratsi, Str. Koryun 2, Yerevan, 0025 Armenia; 8 Department of Prosthodontics, School of Dentistry, Tehran University of Medical Sciences, North Karegar St, Tehran, Iran

**Keywords:** Interleukin-17, Interleukin-23, Gingival crevicular fluid, Periodontitis, Biomarkers, Systematic review

## Abstract

**Background:**

The IL-23/IL-17 axis plays an important role in the immunopathogenesis of periodontal disease. A systematic review was conducted to synthesize all research reporting on the levels of the IL-23/IL-17 axis in gingival crevicular fluid (GCF) from subjects with gingivits, and periodontitis, compared to healthy controls.

**Methods:**

The protocol followed the PRISMA, and Cochrane guidelines, and was registered with the Open Science Framework (OSF): 10.17605/OSF.IO/7495V. A search was conducted in the electronic databases PubMed/MEDLINE, Scopus, Google Schoolar, and Cochrane from November 15th, 2005, to May 10th, 2023. The quality of the studies was assessed using the JBI tool for cross-sectional studies.

**Results:**

The search strategy provided a total of 2,098 articles, of which 12 investigations met the inclusion criteria. The total number of patients studied was 537, of which 337 represented the case group (subjects with gingivitis, and chronic periodontitis), and 200 represented the control group (periodontally healthy subjects). The ages of the patients ranged from 20 to 50 years, with a mean (SD) of 36,6 ± 4,2, of which 47% were men, and 53% were women. 75% of the investigations collected GCF samples with absorbent paper strips, and analyzed cytokine IL-17 levels individually. In addition, qualitative analysis revealed that there are differences between IL-23/IL-17 axis levels in subjects with chronic periodontitis, gingivitis and healthy controls.

**Conclusions:**

Thus, IL-23/IL-17 axis levels could be used in the future as a diagnostic tool to distinguish between periodontal diseases.

## Introduction

Periodontal disease is an umbrella term for a group of diseases that affect the supporting tissues of the teeth (gingiva, root cementum, periodontal ligament and alveolar bone) [1]. The two most common forms of clinical presentation are gingivitis and periodontitis [2]. Gingivitis involves inflammation of the gingiva without apparent changes in clinical attachment levels. In fact, almost half of the world’s population suffers from this condition [3], and if gingivitis is not resolved, a part of gingivitis progresses and gives rise to periodontitis, which corresponds to an inflammatory and destructive process of the periodontium [4]. Both conditions occur in response to a dysbiotic polymicrobial challenge with a high prevalence of periodontopathogens such as *Porphyromonas gingivalis*, *Tannerella forsythia*, and *Treponema denticola*, accompanied by an aberrant immune response, in a genetically susceptible host [5]. Periodontitis is the sixth most common osteolytic disease affecting humans [6] and currently has a prevalence of 62.3%, and in its most severe form can affect up to 23.6% of the world’s population [7]. For didactic purposes, periodontitis is classified into chronic and aggressive [8, 9].

Periodontal probing and radiographic evaluation are considered the gold standard for establishing the diagnosis of periodontal disease [10]. However, clinical parameters and radiographic diagnosis only represent the sequelae of a previous bacterial challenge, and alone do not assess the onset and progression of periodontal destructive changes [11]. In this sense, biomarkers are host-derived molecules whose main purpose is to identify the state of health or disease [12]. In the oral cavity, they can be detected in saliva, tissue biopsies, supra- and subgingival plaque, peri-implant gingival crevicular fluid (PICF), as well as in gingival crevicular fluid (GCF) [13]. The GCF consists of a complex mixture of serum-derived substances such as leukocytes and their products (cytokines, chemokines, enzymes), inorganic ions, structural cells of the periodontium and oral bacteria [14–18].

Inflammatory mediators are the most commonly studied type of biomarkers in periodontal diseases, and their importance lies in the fact that these molecules reflect the dynamics of inflammation, highlighting the innate and adaptive immune activity of the host [19] in response to microbial-associated molecular patterns (MAMPS) in the proinflammatory microenvironment of the gingival sulcus [20]. Among them, interleukin-23 (IL-23), and interleukin-17 (IL-17), have been investigated individually and together to elucidate their role in the pathogenesis of periodontal diseases [21]. In this context, one of the main functions of IL-23 is to regulate the differentiation of CD4 and CD8 (+) T naive cells to T helper 17 (Th17) cells [22], in turn Th17 cells produce tumor necrosis factor alpha (TNF-α), which promotes the development of myeloid cells, induces osteoclastic activity, inhibits osteoblastic activity and up-regulates the production of other cytokines/chemokines such as interleukin-6 (IL-6), interleukin-1 beta (IL-1β), interleukin-8 (IL-8), and ligan 1 (C-X3-C motif) (CX3CL1), creating sustained feedback loop, which enhances disease development [23]. IL-17 regulates the migration of neutrophils which phagocytize bacteria, release their extracellular traps (NETs) and with it lysosomal enzymes to fight pathogens [24, 25]. In addition, IL-17 acts synergistically with TNF-α and IL-1 to induce the release of receptor activator of NF-κB ligand (RANKL) that binds with its receptor RANK on the surface of preosteoclasts, producing osteoclastogenesis and initiating the process of bone destruction in periodontitis [26, 27].

Numerous studies have reported differences in IL-23/IL-17 axis levels in GCF [28–39] and in other biological samples such as gingival tissue [40], serum [22] and saliva [41] from subjects with gingivitis and periodontitis, suggesting that this axis is involved in the progression and severity of periodontal diseases [21]. Moreover, it has been shown that, IL-1β is considered a remarkable inflammatory biomarker in the development and progression of gingivitis [42] and periodontitis [43]. While, on the other hand, a close association between elevated TNF-α levels in GCF with periodontal disease has also been demonstrated, supporting its use as a potential biomarker for its diagnosis [44]. However, to date, it has not been determined whether the IL-23/IL-17 axis could be a practical and accurate indicator based on GCF analysis to distinguish between periodontal diseases.

Therefore, the objectives of the present study were:

1. To perform a comprehensive systematic review of the literature and compile the available evidence on IL-23/IL-17 axis levels in GCF of subjects with periodontal disease.
2. To identify whether IL-23/IL-17 axis can be used as a diagnostic tool to distinguish between periodontal diseases.

## Materials and methods

### Protocol registration

The present study followed the Preferred Reporting Items for Systematic Review and Meta-Analysis (PRISMA), [45] and Cochrane Handbook for Systematic Reviews guidelines [46]. The protocol was recorded with the OSF enrollment (Registration DOI: 10.17605/OSF.IO/7495V).

### PICO focus question

The central question was formulated considering the PICO elements (Population, Intervention, Comparison, and Outcome).

- **P**: Subjects with periodontal disease (gingivitis, and chronic periodontitis).
- **I**: IL-23, and IL-17 levels in GCF can be used to differentiate between healthy, gingivitis, chronic periodontitis subjects.
- **C**: Changes in IL-23, and IL-17 levels in GCF of healthy, gingivitis, and chronic periodontitis subjects.
- **O**: There is a difference in the level of IL-23, and IL-17 between (1) healthy versus gingivitis; (2) gingivitis versus chronic periodontitis; (3) healthy versus chronic periodontitis.

The question was the following: Are there differences in IL-23 and IL-17 levels in GCF between chronic periodontitis, gingivitis and periodontally healthy subjects?

### Eligibility criteria

#### Inclusion criteria

For this systematic review, inclusion criteria were as follows:

- Original cross-sectional, and longitudinal clinical studies that analyzing IL-23, and IL-17 levels in GCF of subjects with gingivitis, and chronic periodontitis diagnosed according to clinical parameters.
- Studies that analyzing the proteins by ELISA technique.
- Studies that included systemically healthy subjects, without any comorbidities. Non-smokers, no antibiotics and/or immunosuppressors, as well as without orthodontic appliances.
- Articles in English language.
- Articles published after 2000.

#### Exclusion criteria

Exclusion criteria were as follows:

- Experimental studies with animal models or cell lines that analyzing the levels of proinflammatory cytokines.
- Quantification of IL-23/IL-17levels in saliva, serum or gingival tissue.
- Unreported exact numbers of cytokines levels.
- Studies that analyzing proteins by other techniques such as western blott, flow cytometry using a bead array system and/or immunoprecipitation.
- Articles in a language other than English.
- Articles published before 2000.

### Search strategy

Two researchers (M.A.A.S and C.G.V) performed a comprehensive electronic search in the following databases: PubMed/MEDLINE, Scopus, Google Schoolar, and Cochrane from November 15th, 2005, to May 10th, 2023, with the main purpose of finding the most relevant articles according to the research topic and that previously met the study criteria. Table 1 shows the search terms used. Additionally, the digital search was complemented with an iterative manual search in journals such as: *Journal of Periodontology*, *Journal of Periodontal Research*, *Journal of Clinical Periodontology*, *Periodontology 2000*, *Journal of Periodontal & Implant Science*, and *International Journal of Periodontics & Restorative Dentistry*.

**Table 1:** The full search strategy used in the pubMed, scopus, google schoolar, and cochrane database

Database	Search Strategy
**PubMed**	((“Interleukin-17“[Mesh]) OR “Interleukin-23“[Mesh]) AND “Gingival Crevicular Fluid“[Mesh]) AND “Periodontitis“[Mesh]))
**Scopus, Google Schoolar, and Cochrane**	TITLE-ABS-KEY (Interleukin-17 OR Interleukin-23 AND Gingival Crevicular Fluid AND Periodontal Disease)

### Screening

**Fig. 1:**
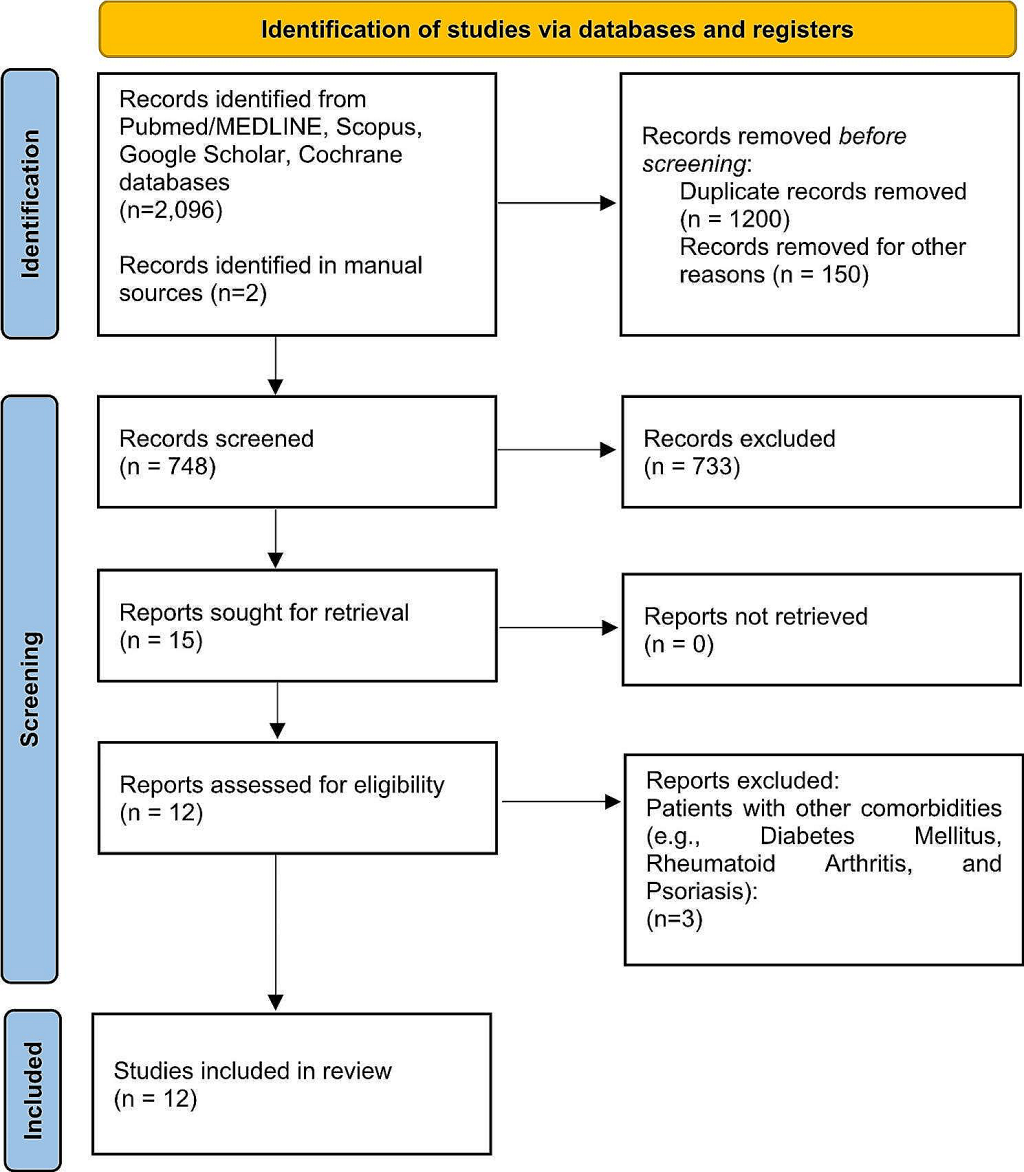
PRISMA flow diagram. PRISMA: Preferred reporting items for systematic and meta-analyses

After retrieval of the articles, the studies were exported to the EndNote reference management tool, facilitating the elimination of duplicates. Next, two investigators (M.A.A.S and C.G.V) independently evaluated the titles and abstracts of each of the articles to determine their suitability for inclusion in the review. Any disagreement between the reviewers involved a third investigator (R.R.M) to resolve the debate, thus excluding irrelevant articles. Finally, the full texts of potentially eligible studies were thoroughly evaluated for inclusion. Cohen’s kappa coefficient was calculated to determine the inter-rater agreement or reproducibility corresponding to the literature selection. The kappa value (κ) was calculated based on the frequency of precise agreements between reviewers. Figure 1 shows the study review process.

### Assessment of quality

The quality of the studies was evaluated with the Joanna Briggs Institute (JBI) critical appraisal tool, which was adapted for cross-sectional studies [47], in the form of a series of question that can be rated as: Yes, No, Unclear, or Not applicable.

The questions were as follows:

1. Were the criteria for inclusion in the sample clearly defined?
2. Were the study subjects and the setting described in detail?
3. Was the exposure measured in a valid and reliable way?
4. Were objective, standard criteria used for measurement of the condition?
5. Was confounding factors identified?
6. Were strategies to ideal with confounding factors stated?
7. Were the outcomes measured in a valid and reliable way?
8. Was appropriate statistical analysis used?

All included articles underwent quality assessment independently by two researchers (R.R.M and J.S.B.R). Finally, the articles were classified in terms of quality, and were placed in three levels: High bias, when the study reached up to 49% of the scores. Moderate bias, when it reached scores of 50 to 69%, and low bias, when it exceeded scores of 70%.

### Data extraction

Data on the eligible articles for this research were extracted by the main reviewer (M.A.A.S), in a customized database in Excel software. All mean values and standard deviations were retrieved from the articles or calculated based on available data. The data extracted were: first author’s name, year, country, study design, title, journal of publication, gender, mean age (SD) of participants, number of cases with periodontal disease and healthy controls, sample size, definition of study groups, GCF sampling, type of biomarkers assessed, type of assay and corresponding kit, the mean levels of cytokines assessed (pg/ml) and their main findings, as well as the quality score for each article.

## Results

### Selection of studies

Initially 2,098 articles were found in the four databases, including PubMed (36 articles were found), Scopus (30 articles were found), Google Scholar (1,990 articles were found), Cochrane (40 articles were found), and manual searching (2 articles were found). Duplicates were removed and, based on title and abstract, the remaining 748 studies were reviewed. After analyzing the full text of the remaining articles, 733 records were excluded as irrelevant. A total of 15 articles were assessed for eligibility, of which 3 studies were excluded because cytokine expression was assessed in patients with other comorbidities (Diabetes mellitus, rheumatoid arthritis, and psoriasis). Therefore, a total of 12 articles were included for the qualitative and quantitative analysis of the present review. The details of study selection are shown in Fig. 1. Cohen’s Kappa coefficient (κ = 0.92) showed almost perfect agreement between reviewers, in the article selection process.

### Description of the studies

Twelve articles with a cross-sectional design were reviewed in this study [28–39]. The total number of subjects studied in the included investigations was 537, of which 337 represented the case group (subjects with gingivitis and chronic periodontitis) and 200 represented the control group (periodontally healthy subjects). The ages of the subjects ranged from 20 to 50 years; the mean ± (SD) age of the subjects studied was 36.6 ± 4.2 years, of which 47% were male and 53% were female. Most of the articles were published after 2014 (8:67%) [28–35]. The oldest study was from 2005 [39], and the most recent from 2022 [28]. Five (41.6%) studies were conducted in India [28–30, 35, 36], two (16.6%) studies in Iran [31, 32], and other studies (8.3%) in Saudi Arabia [33], Japan [34], Egypt [37], Turkey [38], and Chile [39]. In addition, the title and journals of publication are shown (Table 2).

**Table 2:** Clinical, and demographic characteristics of the subjects included in the study

Author/Year	Country	Study design	n (HC,PD)		n (Total)	Gender F^e^/M^a^	Age (mean or range)	Definition of groups study
Nair et al., 2022 [28]	India	Cross-sectional	30	60	90	45^e^ 45^a^	23(2.9)	CP = GI score of > 1, PD of ≥ 5 mm, CAL of ≥ 3 mm, and RBL G = GI score of > 1, PD of ≤ 4 mm and absence of CAL and RBL HC = GI score of 0, PD of ≤ 3 mm and absence of CAL and RBL
Wankhede, and Dhadse, 2022 [29]	India	Cross-sectional	15	15	30	NI	37.5(1.0)	CP = PD ≥ 4 mm, and CAL ≥ 4 mm HC = PD < 3mm, without RBL
Nainee et al., 2020 [30]	India	Cross-sectional	25	50	75	NI	41.3(8.4)	CP = GI > 1, PD ≥ 4 mm, CAL ≥ 3 mm G = GI > 1, PD ≤ 3 mm, no CAL HC = No clinical signs of gingival inflammation, absence of BOP, PD ≤ 3 mm, no CAL
Sadeghi et al., 2018 [31]	Iran	Cross-sectional	10	12	22	11^e^ 9^a^	37.9(7.8)	CP = PD ≥ 5 mm, CAL ≥ 3 mm and BOP HC = Without BOP, gingival inflammation, CAL and PD
Kalate et al., 2018 [32]	Iran	Cross-sectional	30	30	60	28^e^ 32^a^	33.9(5.5)	CP = PD ≥ 5 mm, CAL ≥ 3 mm, presence of BOP, and gingival discoloration HC = PD < 3 mm, and no clinical symptoms of gingival inflammation
Althebeti et al., 2018 [33]	Saudi Arabia	Cross-sectional	10	20	30	30^e^	20–40	CP = PD ≥ 5 mm, and CAL ≥ 3 mm G = Generalized gingival inflammation with BOP, and no CAL HC = Clinically healthy gingiva with IP 0, CAL 0, and PD ≤ 3 mm
Mitani et al., 2015 [34]	Japan	Cross-sectional	10	16	26	15^e^ 14^a^	48.5(1.9)	CP = PD ≥ 5 mm, CAL ≥ 6 mm and RBL HC = PD ≤ 3 mm, BoP%≤10% without RBL
Himani et al., 2014 [35]	India	Cross-sectional	17	33	50	25^e^ 25^a^	36.9(3.2)	CP = GI ≥ 2, CAL ≥ 8 mm, PD ≥ 5 mm G = GI > 1, CAL0mm, PD ≤ 3 mm HC = GI ≤ 1, CAL0mm, PD ≤ 3 mm
Nagireddy et al., 2013 [36]	India	Cross-sectional	10	20	30	NI	20–50	CP = PD > 5 mm, and RBL HC = Clinically healthy periodontium, and no evidence of RBL
Shaker et al., 2012 [37]	Egypt	Cross-sectional	15	25	40	17^e^ 23^a^	32.6(2.6)	CP = Subjects were > 35 years of age with PD and CAL > 5 mm HC = Subjects were > 20 years of age and had clinically healthy gingival with PI = 0, GI = 0, PD and CAL ≤ 3 mm
Ay et al., 2009 [38]	Turkey	Cross-sectional	20	40	60	26^e^ 34^a^	37.6(1.0)	CP = Subjects with RBL, PD, and CAL > 4 mm HC = Subjects had no evidence of CAL and RBL
Vernal et al., 2005 [39]	Chile	Cross-sectional	8	16	24	17^e^ 7^a^	37.4(8.0)	CP = Subjects with PD ≥ 5 mm, CAL ≥ 3 mm, and extensive RBL HC = Subjects with absence of CAL
Summary of variables included in the study ◊			200	337	537	214^e^/189^a^	36.6(4.2)	

*Abbreviations* NI Not information, PD Periodontal disease, CP Chronic periodontitis, G Gingivitis, HC Healthy control, PI Plaque index, GI Gingival index, PD Probing deep, CAL Clinical attachment level, BOP Bleeding on probing, RBL Radiographic bone loss

Nine (75%) studies collected GCF samples with absorbent paper strips [30–35, 37–39] and 3 (25%) studies reported GCF collection using volumetric microcapillary pipettes [28, 29, 36]. Among the 12 included studies, 4 (33.3%) used ELISA kits without brand specification [30, 32–34], two (16.6%) used R&D Systems ELISA kit [36, 39], two others (16.6%) used BioSource ELISA kit [37, 38], others (8.3%) used RayBiotech [28], Diaclone [29], Bender Med Systems [31] and eBioscience [35]. Most of the studies (75%) analyzed cytokine IL-17 levels individually [28–30, 32, 34, 36–39], whereas, 2 others (17%) analyzed IL-23 levels [33, 35] and only one (8%) analyzed the IL-23/IL-17 axis together [31] (Table 3).

**Table 3:** Characteristics of the biomarkers assessed

Author/Year	GCF Sampling	Biomarker Assessment	Type of Assay/ Kit	Biomarkers Mean Value/(SD)	Main Findings
Nair et al., 2022 [28]	Volumetric microcapillary pipette extracrevicular	IL-17	ELISA (RayBiotech)	IL-17 (pg/mL) HC: 11.56(0.99)* G: 19.27(2.78)* CP: 99.67(18.85)**	↑ Subjects with CP, and G In CP, IL-17 was + correlated with CAL (*p < 0.05)
Wankhede, and Dhadse, 2022 [29]	Volumetric microcapillary pipette extracrevicular	IL-17	ELISA (Diaclone)	IL-17 (pg/mL) HC: 0.64(0.23)* CP: 1.96(1.71)**	↑ Subjects with CP In CP, IL-17 was + correlated with CAL (*p < 0.05)
Nainee et al., 2020 [30]	Paper points	IL-17	ELISA	IL-17 (pg/mL) HC: 8.19(7.61)* G: 122.35(172.11)* CP: 178.71(199.32)**	↑ Subjects with CP, and G In G, and CP, IL-17 was + correlated with GI, PD, and CAL (*p < 0.001)
Sadeghi et al., 2018 [31]	Paper strips	IL-17, IL-23	ELISA (Bender Med Systems)	IL-17 (pg/mL) HC: 22.81(23.63)** CP: 1.46(1.20)* IL-23 (pg/mL) HC: 186.55(183.51)** CP: 91.21(1.0)*	↓ Subjects with CP Also, a + correlation was found between the levels of both cytokines, (*p < 0.001), but there were no significant correlations with periodontal clinical parameters (*p > 0.001)
Kalate et al., 2018 [32]	Paper strips	IL-17	ELISA	IL-17 (pg/mL) HC: 38.18(11.23)* CP: 53.46(45)**	↑ Subjects with CP (*p < 0.001)
Althebeti et al., 2018 [33]	Paper strips	IL-23	ELISA	IL-23 (pg/mL) HC: 27.26(19.74)* G: 42.57(4.47)* CP: 107.01(53.50)**	↑ Subjects with P, and G IL-23 was + correlated with PI in CP (*p < 0.001)
Mitani et al., 2015 [34]	Paper strips	IL-17	ELISA	IL-17 (pg/mL) HC: 6.51(4.71)* CP: 23.37(6.62)**	↑ Subjects with CP In CP, IL-17 was + correlated with PD, and CAL (*p < 0.05)
Himani et al., 2014 [35]	Paper strips	IL-23	ELISA (eBioscince)	IL-23 (pg/mL) HC: 0.66(0.32)* G: 4.01(1.3)* CP: 10.19(4.8)**	↑ Subjects with CP, and G (*p < 0.05)
Nagireddy et al., 2013 [36]	Volumetric microcapillary pipette extracrevicular	IL-17	ELISA (Quantikine® R&D systems)	IL-17 (pg/mL) HC: 36.87(10.50)* CP: 54.75(12.35)**	↑ Subjects with CP In CP, IL-17 was + correlated with PD (*p < 0.001)
Shaker et al., 2012 [37]	Paper strips	IL-17	ELISA (BioSource)	IL-17 (pg) HC: 30.5(3.8)* CP: 37.4(8.8)**	↑ Subjects with CP (*p < 0.001)
Ay et al., 2009 [38]	Paper strips	IL-17	ELISA (BioSource)	IL-17 (pg) HC: 14.05(1.0) CP: 11.62(1.0)	No significan differences were found among the groups in the total amount of IL-17
Vernal et al., 2005 [39]	Paper strips	IL-17	ELISA (Quantikine® R&D Systems)	IL-17 (pg) HC: 35.6(2.4)* CP: 45.9(17.4)**	↑ Subjects with CP (*p < 0.001)

*Abbreviations* P Periodontitis, G Gingivitis, HC Healthy control, PI Plaque index, PD Probing deep, CAL Clinical attachment level, BOP Bleeding on probing, IL-17 Interleukin-17, IL-23 Interleukin-23, ELISA Enzyme-linked immunosorbent assay

### Assessment of study quality and risk of bias

The Joanna Briggs Institute (JBI) checklist was used to assess the quality of the cross-sectional studies. According to the established criteria, all articles achieved scores of 100 [28–39], resulting in a low risk of bias in all selected studies (Table 4).

**Table 4:** Quality assessment for clinical studies included in the presente review according to the critical appraisal tools of JBI Scale for analytical cross-sectional studies

Authors	(1)	(2)	(3)	(4)	(5)	(6)	(7)	(8)	Overall score and quality
Nair et al., [28]	Y	Y	Y	Y	Y	Y	Y	Y	100
Wankhede, and Dhadse, [29]	Y	Y	Y	Y	Y	Y	Y	Y	100
Nainee et al., 2020 [30]	Y	Y	Y	Y	Y	Y	Y	Y	100
Sadeghi et al., [31]	Y	Y	Y	Y	Y	Y	Y	Y	100
Kalate et al., [32]	Y	Y	Y	Y	Y	Y	Y	Y	100
Althebeti et al., [33]	Y	Y	Y	Y	Y	Y	Y	Y	100
Mitani et al., [34]	Y	Y	Y	Y	Y	Y	Y	Y	100
Himani et al., [35]	Y	Y	Y	Y	Y	Y	Y	Y	100
Nagireddy et al., [36]	Y	Y	Y	Y	Y	Y	Y	Y	100
Shaker et al., [37]	Y	Y	Y	Y	Y	Y	Y	Y	100
Ay et al., [38]	Y	Y	Y	Y	Y	Y	Y	Y	100
Vernal et al., [39]	Y	Y	Y	Y	Y	Y	Y	Y	100

Question (Q); N/A, not aplicable; Y, yes; U, unclear. (1)Were the criteria for inclusion in the sample clearly defined? (2)Were the study subjects and the setting described in detail? (3)Was the exposure measured in a valid and reliable way? (4)Were objective, standard criteria used for measurement of the condition? (5)Was confounding factors identified? (6)Were strategies to ideal with confounding factors stated? (7)Were the outcomes measured in a valid and reliable way? (8)Was appropriate statistical analysis used?

## Discussion

A systematic review was conducted, which evaluated IL-23/IL-17 axis levels in GCF of subjects with chronic periodontitis, gingivitis, and healthy controls, from 12 independent cross-sectional studies corresponding to seven different countries.

In the gingival sulcus, the presence of a dysbiotic microbiome, which disrupts host immune responses, constitutes the main cause of the initiation, establishment and progression of inflammation (gingivitis), and subsequent destruction of tooth-supporting tissues (periodontitis) [48]. On the one hand, innate immunity represents the initial response of the host, constituting the first line of defense against invasion by pathogens, whereas, adaptive immunity represents the response following specific exposure to a given antigen, and is mediated mainly by T cells; which participate in cellular immunity against intracellular pathogens and, B cells; which participate in humoral immunity, through the production of antibodies directed against extracellular pathogens and microbial toxins [49]. Definitely, the interaction between both systems is quite complex, but very important to regulate and maintain tissue homeostasis [50].

In relation to cellular immunity, and immediately after CD4 and CD8 (+) T naive cells have been exposed to a particular antigen and/or cytokines present in the microenvironment, these cells proliferate, and differentiate into effector cells, which include cytotoxic T lymphocytes (CTL) and T helper (Th) cells [51]. In this context, some proinflammatory cytokines such as IL-1β, IL-6 and IL-23, as well as, the transcription factors signal transducer and activator of transcription 3 (STAT3), and retinoid-related orphan receptor-γt (RORγt), participate in the differentiation of CD4 (+) T naive cells into Th17 cells [52]. Actually, what happens is that, transforming growth factor beta (TGF-β), IL-1β and IL-6 inhibit forkhead box P3 (FOXP3), which is a negative regulator of Th17 cells and simultaneously activate RORγt initiating the Th17 cell differentiation cascade [53]. When RORγt is absent, RORα gives rise to this mechanism [54]. Therefore, the presence of these transcription factors is quite important for the generation of the Th17 subset [55].

**Fig. 2:**
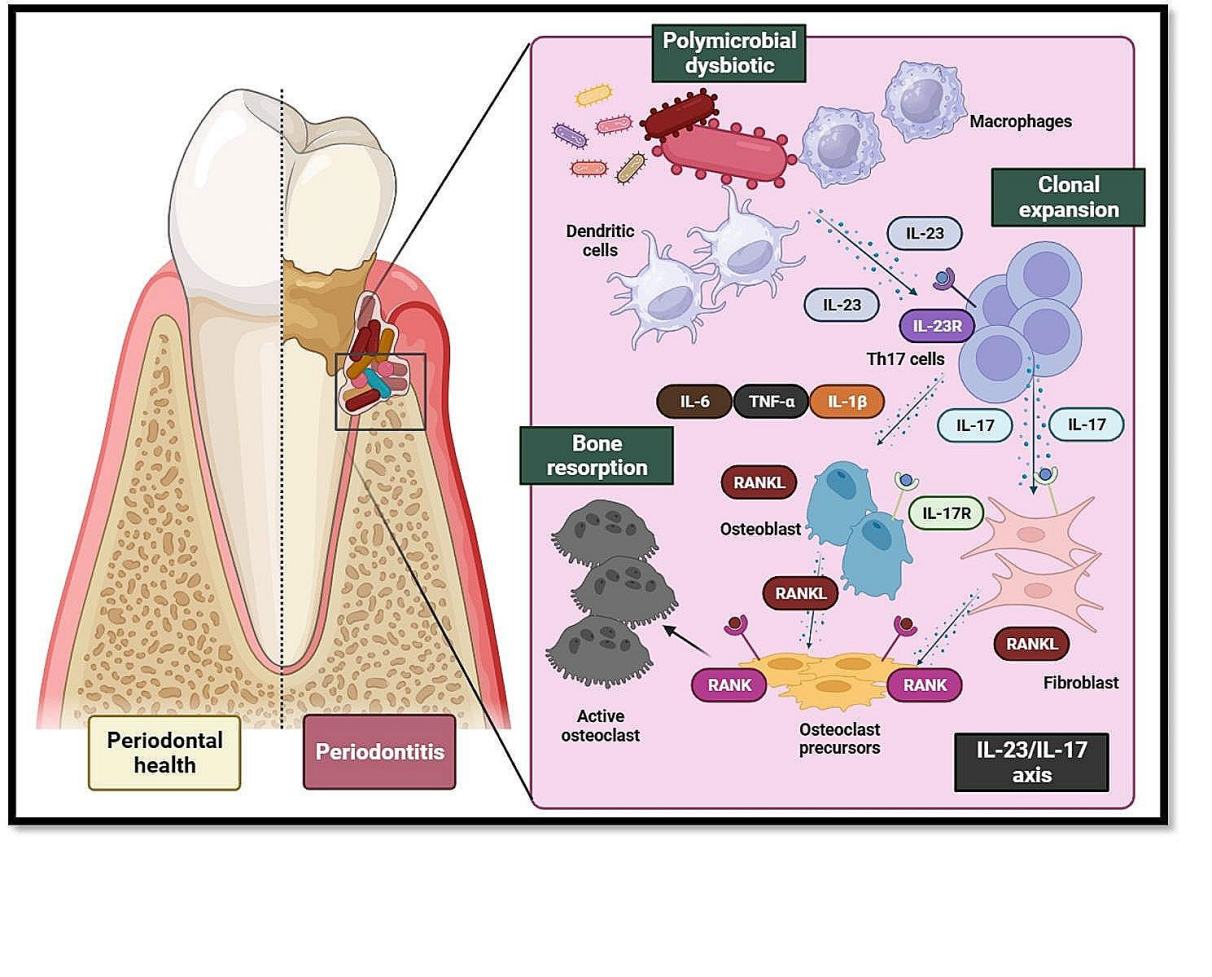
Role of the IL-23/IL-17 axis in periodontitis

Specifically, in the IL-23/IL-17 axis, IL-23 is first secreted by antigen presenting cells such as dendritic cells, and macrophages in response to polymicrobial challenge as pathogen associated molecular patterns, and damage-associated molecular patterns (PAMPS, DAMPS respectively) as well as MAMPS. Subsequently, IL-23 interacts and binds with its receptor (IL-23R) present on the cell membrane of Th17, which on the one hand, up-regulates RORγt expression through STAT3; creating a feedback loop sustained by IL-23. And on the other it also induces the production of other proinflammatory cytokines such as some members of the tumor necrosis factor superfamily (TNF-α and RANKL), interleukin-22 (IL-22), and IL-17, contributing to the bone resorption process [56]. Importantly, IL-23 signaling alone does not induce the development of Th17 cells from CD4 (+) T naive cells, as its receptor is expressed after differentiation into Th17 cells is initiated [57] (Fig. 2).

In fact, it is presumed that these molecules could be used as complementary tools to clinical parameters to diagnose and assess the degree of progression of periodontal diseases [58, 59].

TNF-α is a proinflammatory and pleiotropic cytokine, whose main function lies in: 1)promotion of myeloid cells, 2)induction of osteoclastic activity; by RANKL-independent paracrine/autocrine signaling or by up-regulation of RANKL, 3)inhibition of osteoblastic activity; by inhibition of the wnt pathway to down-regulate osteoblast function and increase their apoptosis and 4) secretion of other cytokines/chemokines and MMPs (MMP-8, MMP-9 and MMP-13) perpetuating the proinflammatory and destructive state in the periodontium [60, 61]. Numerous studies have shown differences in TNF-α levels in saliva, serum, tissue biopsies, PICF and GCF of subjects with periodontal disease [62–66]. In fact, it is currently considered a potential inflammatory biomarker to distinguish between periodontal (gingivitis and chronic periodontitis) and peri-implant (mucositis and peri-implantitis) diseases [44, 67].

In relation to IL-22, there is little evidence of its role in the pathogenesis of periodontal disease, however, it is known to play an important role in mucosal immunity [68]. In this regard, it is documented that IL-22 does not act directly on immune cells, but acts on cells of tissues such as skin and mucosa. Thus, it has been shown that, in keratinocytes IL-22 induces the production of antimicrobial peptides such as human beta defensin-2 (HBD-2) which plays a key role in host defense against infection. In fact, increased levels of IL-22 and HBD-2 in GCF, as well as, a positive correlation between both proteins with periodontal clinical parameters; plaque index (PI), gingival index (GI), probing deep (PD), and clinical attachment level (CAL) have been reported in subjects with gingivitis and chronic periodontitis, however, to distinguish between both conditions, subjects with chronic periodontitis presented a significant increase of IL-22 and HBD-2 levels in GCF compared to subjects with gingivitis [69]. On the other hand, in individuals with chronic periodontitis and psoriasis, a trend of increased levels of this cytokine has also been reported compared to periodontally healthy individuals with psoriasis [70]. These findings in the literature could be explained by the characteristic polymicrobial dysbiosis in subjects with periodontal disease that stimulates the inflammatory response and thus the large repertoire of Th17 cells, with subsequent production of proinflammatory cytokines that produce osteoclastogenesis [21, 40].

The role of the IL-23/IL-17 axis has been demonstrated in other systemic diseases such as rheumatoid arthritis [71], cancer [72], psoriasis [73], kidney disease [74], inflammatory bowel disease [75] and cardiovascular diseases [76]. In addition, several studies have explored the role of the IL-23/IL-17 axis in all types of periodontal diseases. These studies have examined the levels of the IL-23/IL-17 axis in different biological samples such as saliva, serum, tissue biopsies, PICF and GCF using some immunoassay methods such as ELISA [22, 28–41] and immunohistochemistry [77], as well as other molecular biology methods such as polymerase chain reaction [78].

The new classification of periodontal and peri-implant diseases proposed by the 2017 global workshop identified three forms of periodontitis; necrotizing periodontal disease, periodontitis as a manifestation of systemic diseases and that encompassed in a single term, as “periodontitis” (referring to chronic and aggressive periodontitis) taking into account the progression and severity of the disease; represented by stages I-IV and grades A-C [79]. In the present review, most of the studies compared individuals with chronic periodontitis with periodontally healthy subjects.

Today, GCF is considered one of the most reliable sources of oral biomarkers, due to its easy availability, with a high potential to reflect health and/or disease status. As mentioned above, there are different collection techniques. In the present study, the use of absorbent paper strips was the method most commonly used by the investigators, followed by the microcapillary pipetting technique. It is important to mention that, both techniques may have some limitations that affect the quantity and quality of the previously collected fluid, and therefore may contribute to the heterogeneity of the data. These changes mainly influence the collection time. In the first technique, a short time of 30 to 60 s is normally required, whereas, in the second technique, it varies between 40 min in healthy sites and 10 min in diseased sites. In both techniques, there is also the possibility of contamination with saliva or blood, which again motivates repeat sampling [80].

Ideally, a biomarker should meet some important criteria, such as validity, should be easy to use and measure, should be affordable, cost-effective, and able to be collected noninvasively. In addition, it should show sensitivity (identification of individuals who actually have the disease) and specificity (those who actually do not have the disease). One of the advantages of using IL-23/IL-17 axis as a diagnostic marker of periodontal disease is due to its ability to be analyzed in different oral fluids and tissues compared to other cytokines/chemokines that are more plasma specific [81].

The results of our study showed an increase in the levels of IL-23/IL-17 axis in GCF of individuals with gingivitis compared to healthy controls, likewise the most important finding of this study showed that the mean level of IL-23/IL-17 axis in GCF of subjects with chronic periodontitis significantly increased compared to gingivitis and periodontally healthy individuals.

Nair et al., [28], Wankhede and Dhadse, [29], Nainee et al., [30], Kalate et al., [32], Althebeti et al., [33], Mitani et al., [34], Himani et al., [ 35], Nagireddy et al., [36], Shaker et al., [37] and Vernal et al., [39] showed in their investigations that the levels of the IL-23/IL-17 axis in GCF were increased and positively correlated with the progress and severity of periodontal disease (GI, PD, CAL). Therefore, scientific evidence suggests that this axis can potentially be considered as biomarkers of inflammation and destruction of periodontal tissues. Also, a positive evaluation was found between the levels of both molecules, which is explained by the fact that IL-23 can produce clonal expansion of Th17 and induce the expression of IL-17 [56]. It is worth mentioning that, only two studies [31, 38] found elevated levels of these cytokines in GCF from periodontally healthy individuals compared to subjects with gingivitis and chronic periodontitis. But this does not necessarily mean that there is a lower production of both cytokines in periodontal diseases. It is recommended to carry out future studies, with a better methodological design, in which individuals who present the different forms of periodontal disease are included, with a larger sample size and where the main cytokines that are involved in the axis can be evaluated. IL-23/IL-17 along with its receptors and isoforms present.

The main limitations of this review were the methodological design of the included cross-sectional studies, so researchers should be encouraged to carry out follow-up studies evaluating changes in the levels of the IL-23/IL-17 axis before and after periodontal therapy; the inclusion of a small number of articles, especially in relation to IL-23, so a meta-analysis was not possible; and a high heterogeneity of the available data, which is given by differences in the included variables, such as GCF sampling, collection time, variation of the periodontal microbiome between individuals, sex, age and systemic inflammatory condition of the body, so the results must be analyzed with great caution.

## Conclusions

The levels of the IL-23/IL-17 axis are increased in GCF of subjects with chronic periodontitis and gingivitis compared to periodontally healthy individuals. Therefore, it could be used as a diagnostic tool to distinguish between periodontal diseases.

## Data Availability

The data supporting this study’s findings are available from the corresponding author upon reasonable request.

## Abbreviations

IL-23: Interleukin-23
IL-17: Interleukin-17
Th17: T helper cells 17
GCF: Gingival crevicular fluid
PICF: Peri-implant gingival crevicular fluid
ELIA: Enzyme-linked immunosorbent assay
MAMPS: Microbial-associated molecular patterns
PAMPS: Pathogen associated molecular patterns
DAMPS: Damage-associated molecular patterns
IL-22: Interleukin-22
IL-6: Interleukin-6
IL-1β: Interleukin-1 beta
TGF-β: Transforming growth factor beta
TNF-α: Tumor necrosis factor- alpha
CX3CL1 Ligan 1: (C-X3-C motif)
IL-8: Interleukin-8
NETs: Neutrophil extracellular traps
MMPs: Matrix metalloproteases
RANKL: Receptor activator of NF-κB ligand
RANK: Receptor activator of NF-κB
CTL: Cytotoxic T lymphocytes
STAT3: Signal transducer and activator of transcription 3
RORγt: Retinoid-related orphan receptor-γt
FOXP3: Forkhead box P3
RORα: Retinoid-related orphan receptor-α
HBD-2: Human beta defensin-2
GI: Gingival index
PI: Plaque index
PD: Probing deep
CAL: Clinical attachment level
